# Internet use, needs and expectations of web-based information and communication in childbearing women with type 1 diabetes

**DOI:** 10.1186/1472-6947-11-49

**Published:** 2011-07-07

**Authors:** Carina Sparud-Lundin, Agneta Ranerup, Marie Berg

**Affiliations:** 1 Institute of Health and Care Sciences, The Sahlgrenska Academy at University of Gothenburg, Box 457, SE-405 30, Gothenburg, Sweden; 2 Department of applied IT, University of Gothenburg, 412 96 Gothenburg, Sweden

## Abstract

**Background:**

In the childbearing period women use the internet both to seek information and as an important source of communication. For women with type 1 diabetes, pregnancy and early motherhood constitute a more complex situation than for women in general. This implies need for support from various professionals and a way of bridging any discontinuity in care would be to develop a website providing complementary social support and information. The objective of this study was to explore internet use, needs, and expectations regarding web-based information and communication in childbearing women with type 1 diabetes.

**Methods:**

Data were collected via a web-based survey with an explorative and descriptive design, in which 105 of 139 eligible mothers with type 1 diabetes and recent childbearing experience participated. The data were analyzed with descriptive and analytical statistics, and open answers with a directed content analysis.

**Results:**

Of the 105 women, 22% never used the internet to search for information concerning pregnancy, childbirth, and parenthood. 12% searched for information every day, 29% one or more times a week, and 38% one or more times a month. Of the women 44% declared themselves to be passive participants on social websites, and 45% to be active participants. 45% had specific expectations of web-based support directed towards childbearing, especially those with higher educational level (*P *= .01). Expectations of instrumental and informational support included an expert-controlled website with reliable, updated, and information focused on childbearing and diabetes, improved access to diabetes care professionals and alternative ways to communicate and to receive childbearing-related support. The women also asked for online technical devices to manage the frequent monitoring of blood glucose during pregnancy. Informal, emotional, and appraisal support from women in similar situations was suggested as a way to provide an arena for belonging instead of creating feelings of alienation.

**Conclusions:**

Our results add important knowledge about the web-based needs of women with type 1 diabetes in relation to childbearing. This user directed study indicates specific areas of development for the provision of effective web-based support that includes facilities for reliable information, interactive support and social networking in this population.

## Background

Today, most women in the childbearing period use the internet both for information and as an important source of communication [1-3]. Access to information via the internet can help to prepare pregnant women before meetings and communication with health professionals, and also to influence decision making [4]. Multiple sources of web support for individuals and defined patient groups can improve health and well-being [5,6]. However, many health professionals do not seem to use the possibility of incorporating internet information in the care of patients in general [7] including pregnant women [1]. This may be due to lacking confidence in reliable sources [8]. Griffiths et al. [9] recommend careful consideration of the reasons for choosing the internet as a venue for interventions, by raising questions about its unique contribution to improving people's health.

Needs and expectations of the internet as a source of knowledge and communication have, as far as we know, not yet been explored in childbearing women with diabetes. This is a high-risk group with respect to maternal, fetal and neonatal outcomes [10,11]. Better glycemic control during pregnancy has been shown to greatly improve outcomes, as a result of intensive medical control and support from health care professionals, and of women's careful daily routines including frequent blood glucose measurement and insulin adjustment [12]. The struggle to obtain optimal glycemic control is demanding for these women, as they are experiencing unfamiliar body responses, stress, worry, insecurity, and unpredictability [13-16]. After childbirth, the mothers are faced with new challenges; including fluctuating blood glucose in both the mother and the newborn at the same time that breastfeeding should be established. Irrespective of different organizational routines in Sweden, there is often a gap in the continuity of care after childbirth, and the mothers often feel alone in a vulnerable situation [17]. Women with diabetes have specific needs during the whole childbearing period [14,16-19], not only for intensive medical consultation, but for social support from health care professionals, relatives, and their peers -- women facing a similar situation [4]. Social support contributes to well-being, and is an interactive process of vital importance in everyday life [4]. Women with diabetes can feel more dependent on their partners when they become mothers [20]. Complementary social support may decrease this dependency and lessen the burden for relatives in everyday life. Facilitating communities with others can provide an important part of social support, and assist in creating stability while managing the transition to motherhood [21].

As part of a larger project aiming to develop a web-based support system for women with type 1 diabetes during pregnancy and early motherhood, the objective of this study was to explore use and needs of web-based information and communication in childbearing women with type 1 diabetes. A presupposition for the project was that a web-based support system might be able to bridge the existing gap between different health care professionals providing care during the childbearing period, and especially during early motherhood. According to Griffiths et al. [9], this kind of project can also provide increased convenience for users in terms of time, mobility, and geography, and promote equal access to quality-assured and evidence-based information.

As noted by Dillon [22], when designing what is intended to be a highly usable web-based support system, a participatory approach can empower users by involving them in the design and artefacts [23,24]. Furthermore, the development of technology support and self-monitoring must take into consideration the users' different degrees of use and experience, as well as how the technology is assimilated within its actual context of use [25]. To that end, studies exploring needs for support can form the basis for development projects [26]. The theoretical basis for the project lies in the theory of social support, developed by House et al. [27], in which social support is assumed to be an interactive process involving four parts: emotional concern, instrumental aid, information and appraisal.

The objective of this study was to explore internet use, needs, and expectations regarding web-based information and communication in childbearing women with type 1 diabetes.

## Methods

The project was approved by the Regional Ethics Board (659-09). The web support system to mothers with type 1 diabetes will be developed through a participatory design including different forms of data collection [24,26]. The present article reports the findings from a web-based survey with an explorative and descriptive design.

### Questionnaire development

After exploring survey design regarding internet use in general and in relation to diabetes and other long-term illnesses, we developed a questionnaire by following recommendations for web-survey design [28]. The questions covered the following aspects: socio demographic factors; use of the internet for information seeking and communication in general, diabetes-related issues and specific questions on needs in relation to childbearing. One open question concerning expectations of web-based support was included in the end of the questionnaire. In order to test functionality of the electronic questionnaire a pilot version of the questionnaire was sent by e-mail to 20 women in the same way as planned for the main investigation. After some minor adjustments, a final version was produced (Additional file 1).

### Sample and participants

The target population was Swedish-speaking women with type 1 diabetes who had given birth to a child in one of two different hospitals in the western region of Sweden during 2007-2009. A total of 160 women were identified from lists obtained from the antenatal clinics in the two hospitals; 21 of these were excluded due to being unable to speak Swedish or to being impossible to reach. The remaining 139 eligible women (87%) were contacted by phone and invited to participate in the study, of which the first 20 women participated in the pilot study. Brief information regarding the purpose of the study was given, and e-mail addresses collected from those women who agreed to participate. These e-mail addresses were stored in a password-protected database.

### Procedure

The questionnaire was distributed via an e-mail in which the women were given a link to an online version hosted by the Webropol online survey service. The e-mail also included information about the purpose of the study, the procedures for participating, the right to decline or cease participation, and an assurance of confidentiality. Consent was considered to have been obtained if the woman completed the questionnaire. One reminder was sent three weeks after the first e-mail. Responses were entered automatically into a database, except the 20 responses from the pilot study and one response obtained by regular mail, which were all manually entered into the database.

### Data analysis

The data from Webropol was converted and transformed into a file suitable for input to the SPSS software package (Chicago, IL); analyses were conducted using version 18.0 of this software. Descriptive statistics were computed to summarize demographic data and categorized responses. The chi square test was used for comparison between proportions of women, conducted at the 5% significance level. Open answers were analyzed with a directed content analysis [29], which is a deductive category application where an existing theory is used to guide and determine the coding scheme [29]. For this purpose, we used the theory of social support developed by House et al. [27], involving four components of social support: emotional support, instrumental support, informational support, and appraisal support. All text from the open answers was sorted into these four predetermined categories of social support, describing variations of needs and expectations regarding web-based support as reported by the women with type 1 diabetes. Examples of these are presented as quotations in the results section.

## Results

### Study group characteristics

A total of 105 women participated in the study (76% of the women contacted). All of the women were living with a partner at the time of the investigation. Thirty-nine (38%) were first-time mothers, while 54 (52%) had two children, eight (8%) had three children, and three had four or more children. Further information on the study group is presented in Table 1.

**Table 1:** Study group characteristics

Variables	Women with type 1 diabetes (n = 105)
**Age**, n (%)	
≤ 30 years	28 (26.7)
31-35 years	40 (38.1)
≥ 36 years	37 (35.3)
**Child's age at time of interview**, n (%)	
< 1 year	21 (20.0)
1-3 years	71 (67.6)
3-5 years	12 (11.4)
< 5 years	1 (1.0)
**Educational level**, n (%)	
Primary school	3 (2.9)
Secondary school	43 (41.3)
University	58 (55.8)
**Current occupation**, n (%)	
Paid work	59 (56.2)
Home work	4 (3.8)
Parental leave	36 (34.3)
Sick leave	7 (6.7)
Unemployed	9 (8.6)
Student	4 (3.8)
**Years with diabetes**, n (%)	
0-9 years	19 (18.3)
10-19 years	42 (40.4)
≥ 20 years	43 (41.3)
**Insulin administration**, n (%)	
Syringe/pen	75 (71.4)
Pump (continuous subcutaneous insulin infusion)	30 (28.6)

Almost all women reported use of the internet to seek information; 81 of 103 women (79%) for issues both in private life and at work, while 21 (20%) only for information in private life. The frequency of this use varied; 38 of 100 women (38%) used it every day, 45 women (45%) one or more times a week, 16 women (16%) one or more times a month, and one woman (1%) just once in the past month. E-mail and social websites were the most common choices for communication, while online chats, blogs and forums were less popular, with about half of the participants reporting no use of social websites at all (see Table 2). Of those using social websites, 46 of 95 (50%) women declared themselves to be passive participants, and 47 (50%) to be active participants. Passive and active participants did not differ in terms of maternal age (*P *= .87), age of youngest child (*P *= .51), number of children (*P *= .12), or educational level (*P *= .53).

**Table 2:** General use of internet communication in women with type 1 diabetes (n = 105)

Frequency	Every day	One or more times a week	One or more times a month	Less often than once a month	Never
Communication n (%)					
**E-mail**	59 (56.2)	36 (34.3)	7 (6.7)	3 (2.9)	0
**Chat** **(MSN, Skype, etc)**	8 (7.8)	10 (9.8)	19 (18.6)	21(20.6)	44 (43.1)
**Blogs, forums**	7 (6.9)	13 (12.9)	12 (11.9)	15 (14.9)	54 (53.5)
**Social websites** **(Facebook, MySpace, etc)**	42 (40.8)	23 (22.3)	14 (13.6)	4 (3.9)	20 (19.4)
**Public authority websites**	3 (2.9)	16 (15.5)	65 (63.1)	15 (14.6)	4 (3.9)

**Figure 1:**
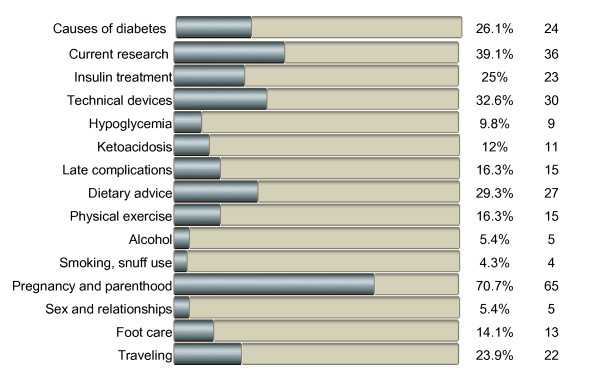
**Areas of information search in relation to general diabetes topics (n = 105)**.

The most common reason for seeking diabetes-related information on the internet was in relation to childbearing (48 of 73 women, 66%) and when planning pregnancy (43 women, 59%). Searching for specific information at the onset of diabetes was reported by 24 women (33%). Figure 1 illustrates the patterns of seeking information on general diabetes-related topics; pregnancy and parenthood was the most frequent area of information search.

**Figure 2:**
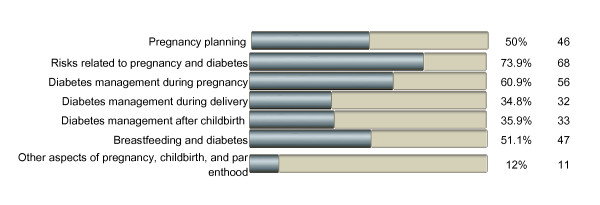
**Areas of information search in relation to diabetes and childbearing (n = 105)**.

In relation to childbearing, 22% (23 of 104) of the women never used the internet to search for information concerning pregnancy, childbirth, and parenthood. Twelve women (12%) searched for information every day, 30 women (29%) one or more times a week, and 39 women (38%) one or more times in the past month. Figure 2 presents the distribution of areas of information search in relation to diabetes and childbearing, showing risks related to pregnancy and diabetes to be the most common reason for information seeking. Of 74 women, 16 (22%) reported that their diabetes care provider had guided them toward finding diabetes-related information on the internet and 22 (29%) had received such guidance from friends, relatives or others, while 36 women (49%) had not received such suggestions from anyone.

The majority of the women (84 of 104, 81%) did not know if their diabetes care provider had a website connected to the clinic, and only seven women (7%) were aware of such a facility. Internet communication with health care professionals in diabetes care was not frequently used (see Table 3). The main methods for such communication were e-mail and a system known as "My Health Contacts", which provides personal services including e-mail contact with an established health care provider.

**Table 3:** Frequency of communication with health care professionals

Frequency	One or more times a week	One or more times a month	Less often than once a month	Never
Communication arena, n (%)				
**E-mail **(n = 100)	1 (1.0)	11 (11.0)	22 (22.0)	66 (66.0)
**Chat **(n = 101)	1 (1.0)	0	3 (3.0)	97 (96.0)
**Blogs, forums **(n = 101)	1 (1.0)	1 (1.0)	4 (4.0)	95 (94.1)
**My Health Contacts **(n = 103)	1 (1.0)	7 (6.8)	16 (15.6)	79 (76.7)

We also asked the women how often they had used internet communication to discuss aspects of pregnancy, childbirth, and parenthood with people other than health care providers. They reported only a limited amount of such communication; 74 women (72%) never used e-mail for this purpose, 87 (85%) never used online chat such as MSN and Skype, and 84 (82%) had not visited diabetes websites with communication facilities. General websites focused on pregnancy, childbirth, and parenthood and also offering communication options were used by 49 women (48%).

### Needs and expectations of web-based support

Forty-seven of 105 women (45%) said that they had felt a great need or quite a great need for web-based support directed towards pregnancy, childbirth, and parenthood. Twenty-eight women (27%) had felt this need to a lesser extent, and 30 women (29%) declared no need of web-based support in these matters. Those who expressed a need did not differ from those who did not in terms of maternal age (*P *= .97), age of youngest child (*P *= .24), number of children (*p *= 0.515), and being a passive/active participant in social websites (*P *= .61). However, women who had completed high school or university declared a greater need for web-based support than those with a lower educational level (*P *= .01).

Via the open question, 48 of the 105 participants expressed the desire for different aspects of web-based support during the childbearing period. The need for formal aid, provided by health care professionals, was almost solely related to instrumental and informational support. This included extended facilities for an expert-controlled link service with relevant websites containing reliable, attractive, updated, and focused information on childbearing and diabetes. The women also expressed a need for improved access to diabetes care professionals, and alternative ways to communicate with those and also to receive childbearing-directed support. Additional requests included online technical devices to manage, transfer, and (together with their care provider) evaluate the frequent monitoring of blood glucose during pregnancy. These expectations are epitomized in the following quotation:

*Instead of visits, it would have been convenient to be able to check with people over the phone or perhaps online, simply to verify blood sugar levels, insulin doses, and the like. Mainly during early pregnancy, when there are no other checks on the child. Also, before the pregnancy is public knowledge, people prefer not to have to go to the hospital for check-ups too often, as it makes them feel as if they are under suspicion at work, which is stressful. In other words, a way of managing and looking after women, particularly in early pregnancy, that is a little more convenient and "discreet", but naturally without imparting a sense that the quality of care is being compromised*.

Requirements for emotional and appraisal support from care providers included a positive attitude in order to alleviate stress and worry over the baby's health due to the diabetes. Informal, emotional, and appraisal support, for example from other women in a similar situation (childbearing women with diabetes), was expected to provide an arena for belonging instead of creating feelings of alienation. The women felt that genuine, well-informed sharing of common experiences would promote the childbearing process. One of them described her experience of this:

*I was part of a forum for pregnant Type-I diabetics on the website "Family life" (spontaneously established). I also met some of the mothers in person, and followed up on them during their second pregnancy too. What felt so important in these contacts was that they felt better understood, and were able to hear about other people in the same situation. Pregnant diabetics go through much more than other mothers, and so it feels even more important to them to find people with whom to share experiences, ideas, and emotions*.

There were also examples of women who preferred live encounters with care providers, and several women had experienced satisfying and extensive support during pregnancy.

## Discussion

As one part of participatory design activities within a larger project to design a web-based support system [24], this study examined the internet habits of childbearing women with type 1 diabetes, focusing on web-based information and communication. We also explored these women's needs and expectations of the internet as a venue for support, since information provision alone is not sufficient. Health care organizations need to take responsibility for understanding how best to meet the need for online information and communication among patients in vulnerable phases of life, as well as within the care system [30]. When designing a directed web-based support system for childbearing women with diabetes, knowledge about user needs might improve the result, and so it was necessary to further investigate the needs of these women.

This study showed that a high proportion of women with type 1 diabetes seek diabetes-related information on the internet, especially before, during, and after pregnancy. This pattern is also found in other studies of internet habits in childbearing women [1,2]. The great majority participated in social websites, and half of these reported active participation. Almost half of the participants stated needs and/or expectations of support, and some women also gave concrete examples of how such support could be designed. As in other studies [2,7], the findings indicate that higher educational level positively influences the need for and use of the internet, suggesting that web-based support might not be equally health-promoting for all women with type 1 diabetes. This can be compared to a study by Larsson [1], which found no association with educational level regarding internet use, areas of information-seeking, and perceptions of reliability of health information on the internet. In our study, pregnancy and parenthood were the most prominent topics for seeking information on the internet, even though many participants also sought information on different diabetes-related issues such as current research, technical devices, dietary advice, and causes of diabetes. Many women obviously initiate this information seeking, as only half of the group had been provided with suggestions of diabetes websites. One way to overcome the barrier of health care providers' suspicion of the quality of web-based information [8] might be to let them become active participants on these websites. Online communication with health care professionals was not common, and slightly less than half of the women had expectations of a web-based support system directed towards pregnancy, childbirth, and parenthood. Although many women were active participants in social websites, few women reported use of online support groups with peers, which is in line with the online health-related activities reported in a national probability sample from the USA [30]. In our study, several women expressed a need for this type of directed online support. The value of peer support has also been confirmed by other studies among women with diabetes [18-21].

As part of the participatory design [24], these findings contribute important details about information and communication needs, and hence provide useful input to further development of web-based support directed at childbearing women with type 1 diabetes. The findings indicate that this group may benefit from specifically designed support from qualified experts. Another relevant area for further investigation is user evaluations of proposed functional design of web-based support including contextual information, provided and controlled by experts, incorporating a social community function, and complementing communication with health care providers.

### Methodological considerations

Although this survey is not by definition an e-survey [31], we applied the Checklist for Reporting Results of Internet E-Surveys (CHERRIES), as it is also valid for surveys distributed by e-mail. A limitation of this kind of distribution is the problem of access to accurate e-mail addresses. Some participants were never reached, as the addresses we had for them were incorrect. Distributing the questionnaire as an e-survey was not an option, because there is no existing website for the target group. The final response rate was considered to be quite satisfactory, according to the somewhat arbitrary limit for estimation of the degree of representativeness [31]. However, one limitation in this study is the lack of ethnic variation among the participants, as the inclusion criteria required that they all be Swedish-speaking. Although some participants originated from countries other than Sweden, all had to understand Swedish properly in order to answer the questionnaire. Due to the underlying objective of designing a web-based support for childbearing women with type 1 diabetes, we also considered it constructive to include open questions that allowed the women to suggest examples of components of web-based support.

## Conclusion

This user directed study contributes with valuable information about the web-based needs of childbearing women with type 1 diabetes. It indicates specific areas of development for the provision of effective web-based support that includes facilities for reliable information, interactive support and social networking in this population.

## Competing interests

The authors declare that they have no competing interests.

## Authors' contributions

CSL and MB designed the study, collected data and wrote initial draft of manuscript.

CSL performed the analysis. AR reviewed and edited the manuscript. All authors have read and approved the final manuscript.

## Pre-publication history

The pre-publication history for this paper can be accessed here:

http://www.biomedcentral.com/1472-6947/11/49/prepub

## Supplementary Material

Additional file 1 **Web questionnaire**. Click here for file
