# Supplementary material for: Internet use, needs and expectations of web-based information and communication in childbearing women with type 1 diabetes
Source: BMC Med Inform Decis Mak. 2011 Jul 7;11:49. doi: 10.1186/1472-6947-11-49 (PMC3141376; doi:10.1186/1472-6947-11-49)
Supplement: Additional file 1 — Web questionnaire. [file 1472-6947-11-49-S1.RTF]

Web-based information and communication among childbearing women with type 1 diabetes

Background information

1. Your age

a) < 25 yrs		b) 26-30 yrs		c) 36-40 yrs		d) >40 yrs	


 2. What is your current living arrangement?

a)	live with the father of my child/children 	
b)	live without a partner
c)	live with a partner other than the father of my child/children
 

3. How many children do you have?

a)	one child
b)	two children
c)	three children
d)	four or more children


4. How old is your youngest child?

a) < 1 year		b) 1-3 yr		c) 3-5 yrs		d) >5 yrs


5. What is the highest level of education you have completed?

a) primary school	b) secondary school	c) university

 
6. What is your current occupation?

a)	working outside the home
b)	working in the home
c)	student	
d)	on parental leave
e)	on sick leave
f)	unemployed


7. For how long have you had diabetes?

a) 0-9 yrs		b) 10-19 yrs		c) 20 yrs or more

8. How do you administer the insulin?

a) with insulin pen		b) with insulin pump


Questions about how you use the internet for information and communication in general


9. Do you use the internet when you need information?

a)	yes, for personal use
b)	yes, for work
c)	both
d)	no (Go to no. 11.)


 10. If you use the internet to search for general information for personal use, about how often do you do so?

a)	every day
b)	several times a week
c)	several times a month
d)	once a month
e)	less than once a month


11. How often do you use the following functions to communicate on the internet?

	every day
	several times a week	several times a month	once a month	less than once a month	never
	
E-mail
							

Chat
(MSN, Skype, etc.)
							
Social communities 
(Facebook, Myspace etc.)
							
Government portals
							
Health care portals


12. If you participate in any social community on the internet, how would you describe your degree of participation? 

a)	passive participant 		b) 	active participant


 Questions about how you use the internet for information and communication about diabetes


13. Do you use the internet to get information on diabetes-related issues? (If no, go to no. 16.)

a)	yes, every day
b)	yes, every week
c)	yes, several times a month
d)	once a  month or less
e)	no, almost never


 14. When did you search for information about diabetes? (Choose as many options as apply.)

a)	at the onset of diabetes 
b)	before visits to the diabetes clinic
c)	before contact with authorities
d)	before discussing my condition with my employer
e)	when planning a pregnancy
f)	in relation to childbirth
g)	other__________________


15. How/from whom did you get information about diabetes websites? (Choose as many options as apply.)

a)	diabetes care provider	
b)	other people with diabetes
c)	the Diabetes Association
d)	relative/friend
e)	received no such information
f)	other


16. What specific diabetes-related information have you searched for?

a)	causes of diabetes
b)	current research
c)	insulin treatment
d)	technical/medical devices
e)	 hypoglycemia
f)	ketoacidosis
g)	late complications
h)	dietary advice
i)	physical activity
j)	alcohol
k)	smoking/snuffing
l)	pregnancy/parenthood
m)	sex and relationships
n)	foot care
o)	travelling
p)	other__________________


17. Does your diabetes care provider have a website with diabetes-related information? (If no, go to no. 19.)

a)	yes	b) 	no	c)	don't know


18. Has the information on your diabetes care provider's website helped you manage diabetes-related issues?

a)	yes, to a great extent
b)	yes, somewhat
c)	no, not particularly
d)	no, not at all


19. If you answered yes to the previous question, how has the website been helpful? (If you answered no, go to no. 20.)

……………………………………………………………………………………………………………………………………………………………………………………………………


20. How often do you use the following functions to communicate with care providers on the internet?		

	every day
	several times a week	several times a month	once a month	less than once a month	never
	
E-mail
							

Chat, blogs
(MSN, Skype, etc.)
							
 
							
”My Health 
Care Contacts”
							
 
  

21. How often do you use the following functions to communicate with others (not care providers) on the internet? 

	every day
	several times a week	several times a month	once a month	less than once a month	never
	
							
E-mail
							
Social communities 
(Facebook, Myspace etc.)
							
Diabetes websites
with chat, blog, and forum features
							


Questions about how you use/have used the internet for information and communication about pregnancy, childbirth and parenthood


22. When you were planning your pregnancy, when you were pregnant or as a new mother, how often did you use the internet to find information about pregnancy, childbirth and parenthood?

a) every day	b) several times a week	c) several times a month			
d) once a month	e) less than once a month	f) never


23. What information did you search for? (Choose as many options as apply.)

a)	diabetes and planning a pregnancy 
b)	pregnancy risks and diabetes  
c)	advice on managing diabetes during pregnancy    
d)	advice on managing diabetes during childbirth
e)	advice on managing diabetes as a new mother
f)	breastfeeding and diabetes
g)	other aspects of pregnancy, childbirth or parenthood
 
	 ………………………………………………………………………..

24. How often have you used/do you use the following functions on the internet to communicate with others about different aspects of pregnancy, childbirth or parenthood?

	every day
	several times a week	several times a month	once a month	less than once a month	never
	
							
E-mail
							

Chat
(MSN, Skype, etc.)
							
Social communities 
focusing on pregnancy, 
childbirth and 
parenthood
(chat, blog, forum)
							
Diabetes websites
with chat, blog, and forum features
							


25. Have you experienced, or are you currently experiencing, the need for a specific website focusing on pregnancy, childbirth or parenthood for those with diabetes?

a)	yes, to a great extent
b)	yes, somewhat
c)	no, not particularly
d)	no, not at all


26. In the space below, describe in your own words what kind of web-based support you would like to get during pregnancy, childbirth and the breastfeeding period.
…………………………………………………………………………………………………
…………………………………………………………………………………………………
…………………………………………………………………………………………………
